# Synthesis and Characterization of Antimicrobial Hydrophobic Polyurethane

**DOI:** 10.3390/ma16124446

**Published:** 2023-06-17

**Authors:** Autumn M. Rudlong, Elizabet Moreno Reyes, Julie M. Goddard

**Affiliations:** Department of Food Science, Cornell University, Ithaca, NY 14853, USA; amr452@cornell.edu (A.M.R.); em827@cornell.edu (E.M.R.)

**Keywords:** antimicrobial polymer, polyurethane, fluoropolymer, food safety, antimicrobial coating, food spoilage

## Abstract

Food borne illness remains a major threat to public health despite new governmental guidelines and industry standards. Cross-contamination of both pathogenic and spoilage bacteria from the manufacturing environment can promote consumer illness and food spoilage. While there is guidance in cleaning and sanitation procedures, manufacturing facilities can develop bacterial harborage sites in hard-to-reach areas. New technologies to eliminate these harborage sites include chemically modified coatings that can improve surface characteristics or incorporate embedded antibacterial compounds. In this article we synthesize a 16 carbon length quaternary ammonium bromide (C16QAB) modified polyurethane and perfluoropolyether (PFPE) copolymer coating with low surface energy and bactericidal properties. The introduction of PFPE to the polyurethane coatings lowered the critical surface tension from 18.07 mN m^−1^ in unmodified polyurethane to 13.14 mN m^−1^ in modified polyurethane. C16QAB + PFPE polyurethane was bactericidal against *Listeria monocytogenes* (>6 log reduction) and *Salmonella enterica* (>3 log reduction) after just eight hours of contact. The combination of low surface tension from the perfluoropolyether and antimicrobial from the quaternary ammonium bromide produced a multifunctional polyurethane coating suitable for coating on non-food contact food production surfaces to prevent survival and persistence of pathogenic and spoilage organisms.

## 1. Introduction

Over 1M foodborne illnesses in the United States are estimated to be attributed to *Salmonella* spp., nontyphoidal and *Listeria monocytogenes* annually [1]. In addition, *Salmonella* spp., nontyphoidal is estimated to be responsible for nearly 20,000 foodborne illness related hospitalizations yearly and both *Salmonella* and *Listeria monocytogenes* related deaths account for 47% of total deaths caused by foodborne illness from pathogenic organisms in the United States [1]. Food processing facilities can harbor pathogenic bacteria including *Salmonella* and *Listeria monocytogenes* in growth niches: areas within a facility that accumulate water and debris [2,3]. These pathogens are known to be resident contaminants in dry and wet facilities and can persist for years even with proper cleaning and sanitation procedures [3].

The FDA and USDA published draft guidance for eliminating the potential for transient and resident pathogens, including *L. monocytogenes*, through proper Current Good Manufacturing Practices [3,4,5,6], yet hard-to-reach growth niches, such as floor drains, remain challenges in the control of microbial persistence and cross-contamination [7,8]. Incorporating hygienic design to reduce bacterial growth niches in newly constructed food processing facilities is an option; however, retrofitting existing facilities is not economically feasible. Following cleaning, food processing facilities apply spray sanitizers typically containing hypochlorous acid, peroxyacetic acid, or quaternary ammonium compounds [9]. Of particular interest to this study, quaternary ammonium compounds (QAC) are antimicrobial agents that contain a cationic nitrogen bound to four carbons, typically containing one long alkyl chain, and one halogen anion. The proposed mechanism of action for QACs is not fully understood but it is generally theorized that the cationic nitrogen will interact with the phospholipid of cell membranes followed by penetration of the alkyl chain, causing cell lysis [10,11]. Alkhalifa et al. explored the bioactivity of different QAC compounds and found that there are three factors which directly affect the potency of QACs [12]. The factors include the length of the hydrophobic tail (in their report, the optimal tail length was found to be 12 carbons), chemistry of tail:QAC bond (alkyl performing better than ester), and the number of cationic residues [12]. QACs have found an interesting place in material and surface science as starting reagents for polymer synthesis and nanoparticles decoration as an example [13]. Previous work has explored the use of quaternary ammonium compounds in polymer synthesis to create antimicrobial materials [9,14,15,16]. Wang et al. created antimicrobial polyurethane with C_12_ QAC chain length with efficacy against Gram-negative and Gram-positive bacteria [16]. Wynne et al. synthesized an antimicrobial polyurethane using alkyl chain lengths of 4, 6, 8, 10, and 18 with C_8_ providing 3 log reduction against Gram-positive and 5 log reduction for Gram-negative [9]. In contrast, Hu et al. synthesized antibacterial polyurethane adhesives via side chain quaternization of a C_4_ alkyl chain which displayed antimicrobial properties [15]. In preliminary work, the authors found optimal antimicrobial activity against Gram-negative and Gram-positive bacteria with synthesized QAS containing carbon chain lengths 16 and 18. These works demonstrate the importance of alkyl chain length in determining the antimicrobial efficacy of a QAC, with optimal chain length varying depending on the organism tested, its Gram stain, and the target end use application.

Food contact and non-contact materials in food processing plants are designed for easy cleaning, chemical resistance, and overall durability. For non-food contact areas such as floors and walls, polyurethane coatings are often applied to protect the surfaces from abrasion while resisting damage from detergents and sanitizers. Polyurethanes consist of hard and soft blocks: the hard blocks, isocyanates, provide durability while the soft blocks, commonly polyether or polyester, provide elasticity. Polyurethanes are synthesized by reaction of isocyanate and hydroxyl groups which provides a great opportunity to make chemical modifications to the polyurethane backbone or incorporate side groups on the polymer via hydroxyl terminated compounds. The surface modification of polyurethane is an expanding area of research in which many researchers focus on reducing the overall surface energy and improving surface hydrophobicity. A relationship between surface free energy and advancing contact angle of a liquid is defined by Young’s equation [17]. An interesting area of chemical surface modifications is fluorinated polymers with the goal being to reduce the overall surface tension of the material and improve hydrophobicity. Fluorinated polymers, commonly known as fluoropolymers, often display hydrophobicity, chemical resistance, durability, and low surface free energy [18,19,20,21]. Fluoropolymers have previously been incorporated into polyurethane with final polymers able to retain the polyurethane durability, mechanical stability, and chemical resistance while introducing hydrophobicity and low surface free energy (6–15 mN m^−1^) [9,18,19,20,21,22,23,24,25,26,27,28,29]. Fluorinated polyols, such as perfluoropolyethers, have been used in surface-modified materials synthesis to impart hydrophobic character due to the large atomic percentage of fluorine. Tonelli and Ajroldi reported the synthesis of polyurethane and perfluoropolyether (PFPE) block co-polymer which possessed characteristics associated with polyurethane including thermal resistance, chemical resistance, and durability in addition to characteristics associated with fluoropolymers including low surface tension [25]. Commercially available PFPE diols have been incorporated into polymers to investigate the potential for improving surface characteristics. Choi et al. reported the synthesis of tri-block methacrylate and Fluorolink E10H diol (PFPE diol) displaying promising surface free energy values (22–25 mN m^−1^) and hydrophobicity [18]. Gu et al. synthesized a polyurethane-poly(isobornyl methacrylate)-perfluoropolyether copolymer with Fluorolink E10H diol and reported a water contact angle of 140° [29].

Multifaceted approaches to combat the presence of bacteria on surfaces, including incorporating antimicrobial compounds [9,15,16,30,31,32,33,34] and surface modifiers [9,20,35] into polymeric coatings, have been previously used to inhibit or eliminate bacterial growth. In this study, a block co-polymer consisting of polyurethane and perfluoropolyether chain extended by quaternary ammonium bromide was synthesized with the aim to produce a low surface energy and antimicrobial polymer coating suitable for preventing microbial contamination of non-food contact growth niches in food processing facilities.

## 2. Materials and Methods

### 2.1. Materials

4,4′-methylenebis(phenyl isocyanate) 98% (MDI), poly(propylene glycol) (725 Da), anhydrous ethylene glycol, anhydrous dimethylformamide 99.8% (DMF), tin(II) 2-ethylhexanoate, potassium phosphate dibasic, and potassium phosphate monobasic were purchased from Millipore Sigma (Burlington, MA, USA). Fluorolink E10H (perfluoropolyether) was graciously donated by Solvay (Bollate, MI, Italy). Type 304 2B finish stainless steel coating panels were purchased from Q-Lab Corporation (Cleveland, OH, USA). Type 304 stainless steel coupons sized 1 cm by 1 cm were fabricated by the Cornell University Laboratory of Atomic and Solid State Physics Machine Shop (Ithaca, NY, USA). The drawdown bar (adjustable thickness gauge, 6 in coating width) for coating was purchased from Paul N. Gardner Company (Pompano Beach, FL, USA). Tryptic Soy Broth (TSB), Plate Count Agar (PCA) and 13 mm × 10 mm borosilicate test tubes were purchased from Thermo Fisher Scientific (Fairlawn, NJ, USA). Furthermore, 1x phosphate buffered saline was purchased from VWR Scientific (Radnor, PA, USA).

### 2.2. Synthesis of Polyurethanes

**Scheme 1:**
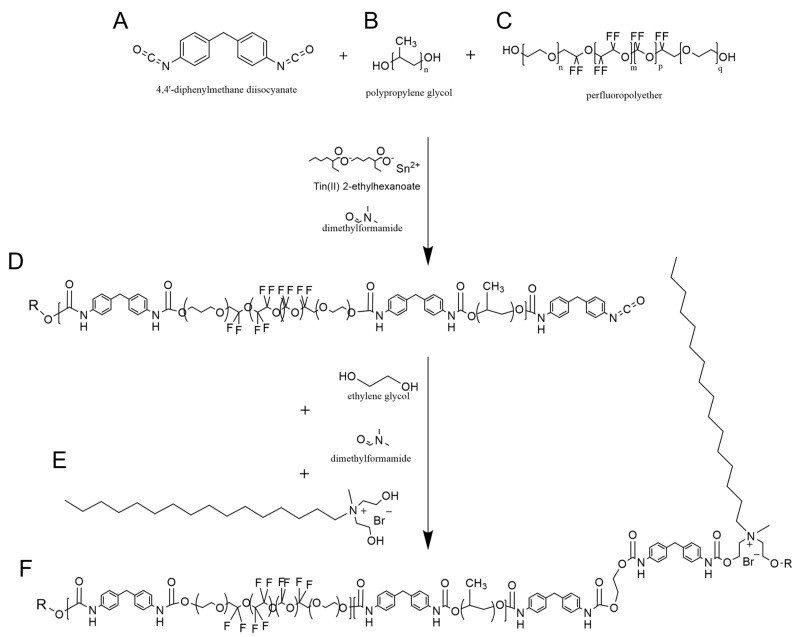
Two-step prepolymer reaction of MDI (**A**), PPG (**B**), and PFPE (**C**) forming the prepolymer (**D**). Chain extender and antimicrobial quaternary ammonium bromide (C16QAB) (**E**) added to form the final polymer C16QAB + PFPE polyurethane (**F**).

Control polyurethane and quaternary ammonium bromide (QAB) modified polyurethane were prepared through a two-step synthesis (Scheme 1, Table 1). In the first step of C16QAB + PFPE polyurethane synthesis, a prepolymer containing 0.09 mmol perfluoropolyether, a fluorinated diol, was produced. Poly(propylene glycol) (6.90 mmol), perfluoropolyether (PFPE) (0.09 mmol) (only in fluorinated polyurethane batches), and anhydrous DMF (16 mL) were placed in a 5-neck reaction vessel fitted with condenser and bubbler, ultra-pure nitrogen, 80 °C silicone oil bath, addition funnel, and overhead stirrer with a paddle attachment. Tin(II) 2-ethylhexanoate was added at 0.5 wt% (0.01 mmol) to catalyze the reaction. Furthermore, 4,4′-methylenebis(phenyl isocyanate) (10.27 mmol for control PU or 10.35 mmol for C16QAB + PFPE PU) was slowly added to the reactor with the overhead stirrer set to 500 rpm to reach a 1:1 diol to isocyanate ratio. The prepolymer reacted for 30 min after which chain extender, ethylene glycol (4.03 mmol), or previously synthesized antimicrobial quaternary ammonium bromide (C16QAB) (0.12 mmol) dissolved in ethylene glycol (3.22 mmol) and anhydrous DMF (8 mL), was added dropwise via addition funnel over 10 min. The polymers were allowed to react for an additional 150 min with the oil bath maintained at 80 °C and stirring at 500 rpm. Following the reaction, polymers were removed from the reaction vessel and vacuum degassed at 10^−2^ mbar for 30 min. After degassing, polyurethane was poured into custom fabricated polytetrafluoroethylene (PTFE) molds measuring 1 cm by 1 cm to create free standing polyurethane films used for FTIR, SEM, and NMR characterization. In addition, polymer was coated to a thickness of 46.67 ± 21 µm (control PU) or 40 ± 5 µm (C16QAB + PFPE PU) onto type 304 stainless steel coupons measuring 1 cm by 1 cm used for XPS characterization and contact angle measurements. All coatings were cured at 80 °C under vacuum at 10^−2^ mbar for 24 h. Coatings were allowed to fully cool to 20 °C prior to removing from the vacuum oven (Binder VDL 23, Binder GmbH, Tuttlingen, Germany) and before any characterization. All coupons were stored in glass dishes within UV protected boxes.

### 2.3. Material Characterization

Samples were sputtercoated with gold (Cressington Sputter Coater 1008auto, Watford, UK) and scanning electron micrographs (SEM) were obtained at 15 kV using a benchtop scanning electron microscope (JCM-6000Plus, Nikon Instruments, Inc., Melville, NY, USA). Two separate films were imaged for each control and C16QAB + PFPE polyurethanes. Micrographs were acquired both on polyurethane surface and cross section. Micrographs were obtained at five different sites on the top surface (center and edges or corners) and at both sides and center of the cross section.Urethane formation was confirmed using attenuated total reflectance Fourier transform infrared (ATR-FTIR) spectroscopy. Spectra were collected using an IRTracer-100 FTIR spectrometer (Shimadzu Scientific Instruments, Inc., Kyoto, Japan) equipped with diamond ATR crystal. Spectra were collected using Happ-Genzel apodization (4 cm^−1^, 32 scans).

Samples were analyzed using a Scienta Omicron ESCA-2SR (Scienta Omicron GmbH, Taunusstein, Germany) with operating pressure ca. 1 × 10^−9^ mbar. Monochromatic Al Kα X-rays (1486.6 eV) were generated at 300 W (15 kV; 20 mA). Analysis spot size was 2 mm in diameter with a 0° photoemission angle and a source to analyzer angle of 54.7°. A hemispherical analyzer determined electron kinetic energy, using a pass energy of 200 eV for wide/survey scans and 50 eV for high resolution scans. All samples were charge neutralized using a low-energy electron flood gun.

^1^H NMR spectra of 25 mg/mL PU and C16QAB + PFPE PU in dimethyl sulfoxide-d_6_ were collected using a Bruker AV500 (Bruker, Billerica, MA, USA). Spectra were analyzed using Mnova (Mestrelab Research, Santiago, Spain).

Water contact angles were measured using the dynamic needle method on an Attension Theta Optical Tensiometer (Biolin Scientific, Stockholm, Sweden). Briefly, advancing water contact angles were determined by depositing a small droplet of water onto the surface followed by inserting the needle into the droplet. The droplet volume was then gradually increased to 5.0 µL at a rate of 0.5 µL/s. The advancing angle was defined as the maximum angle prior to an increase in droplet baseline dimension [35]. The receding water contact angle was determined by withdrawing water from the droplet at a rate of 0.5 µL/s. Receding angles were defined as the minimum angle prior to the recession of the droplet baseline [35]. Contact angle hysteresis was determined by subtracting the receding angle from the advancing angle.

Critical surface tension of the synthesized coatings as well as uncoated stainless steel were calculated using the Zisman plot method [26,27,36,37]. Briefly, four liquids with known surface tensions were individually deposited onto the material surface at a rate of 0.5 µL/s and the advancing angle was taken at the maximum angle prior to the advance in droplet baseline. The cosine of each advancing liquid contact (θ) was calculated and plotted against the known surface tension of the corresponding liquid. Data were fit to a non-linear quadradic model and a replicates test was performed to verify model adequacy. The quadradic equation associated with the line was used to determine material critical surface tension (at cos θ = 1). Liquids used to determine surface tension included deionized water, ethylene glycol, glycerol, and hexane. Quadruplicate measurements were taken for each liquid on each surface.

### 2.4. Antimicrobial Efficacy

The antimicrobial efficacy of control and C16QAB + PFPE modified polyurethanes was characterized using modification of the standardized method ASTM E2149-20, determining the antimicrobial activity of antimicrobial agents under dynamic contact conditions [38]. After synthesis and degassing, 250 µL aliquots of polymer were deposited into sterile 13 × 100 mm borosilicate glass tubes. Coated tubes were placed in a vacuum oven for 24 h at 80 °C to cure the polymer on the bottom of the tubes for a final coating surface area of 1.64 cm^2^. The surface to volume ratio for this study was kept consistent with ASTM E2149-20 at 0.516 cm^2^ coating surface area/mL bacterial culture. All tubes were capped with ethanol-sterilized plastic tube caps after curing and stored in a UV protected desiccator. Additional tubes for a bacterial growth control were autoclaved at 121 °C for 20 min followed by capping with ethanol-sterilized plastic tube caps. *Salmonella enterica* serovar Typhimurium (ATCC 14028) was purchased from American Type Culture Collection (ATCC, Manassas, VA, USA) and *Listeria monocytogenes* was provided by the Food Safety Lab (Cornell University, Ithaca, NY, USA). Overnight bacterial cultures were prepared in 40 g L^−1^ tryptic soy broth (TSB) and grown at 37 °C while shaking at 90 rpm for 16–18 h. The overnight cultures were adjusted to 1.5 × 10^8^ CFU mL^−1^ based on optical density at 600 nm by adding sterile 0.3 mM KH_2_PO_4_ buffer according to a preliminary bacterial growth curve performed in TSB. A bacterial working solution with a concentration of 1.5 × 10^5^ CFU mL^−1^ was prepared using the adjusted bacterial overnight cultures and sterile 0.3 mM KH_2_PO_4_ buffer. Tubes coated in control and C16QAB + PFPE-modified polyurethane along with blank tubes containing no coating were filled with 3 mL of bacterial working solution, capped, and incubated for 8 h at 37 °C while shaken orbitally at ~175 rpm. Bacterial contact time (8 h) was selected to represent a short food industry production shift, or the minimal time between routine cleanings. After 8 h incubation, bacterial suspensions were diluted in sterile PBS and plated on plate count agar (PCA). To lower the limit of detection to 3 CFU ml^−1^, 1 mL of bacterial suspensions was plated directly from the experimental tubes onto PCA plates (333 µL per plate) [39].

### 2.5. Statistics

ATR-FTIR spectra were acquired from four different coating coupons with characteristic band analysis performed using OriginPro 2019 (OriginLab Corporation, Northampton, MA, USA) and KnowItAll Informatics System 2023 (BioRad Laboratories, Hercules, CA, USA). Spectra displayed in figures were randomly chosen by assigning numbers to files (1–4) and using a random number generator to select the spectra. Additional spectra not displayed are available upon request. Surface wettability and surface free energy were performed in quadruplicate on two independently synthesized batches. Antimicrobial efficacy experiments were performed in triplicate with two independently prepared bacterial cultures. Statistical differences between samples were analyzed using one-way analysis of variance (ANOVA) (*p* < 0.05) with Tukey’s HSD multiple comparisons (*p* < 0.05) and line fitting and replicates test using GraphPad Prism version 7.05 (GraphPad Software, San Diego, CA, USA). For surface hydrophobicity, technical replicates (n = 4) were averaged and the experiment was repeated providing two independent averages which were used for ANOVA analysis. For antimicrobial efficacy, plating replicates (n = 2) were averaged to create each technical replicate (n = 3) and this experiment was repeated with a new bacterial sample from the same stock providing two independent averages which were used for ANOVA analysis.

## 3. Results and Discussion

### 3.1. Synthesis

Polyurethane containing 3 wt% commercially available perfluoropolyether and previously synthesized quaternary ammonium bromide was synthesized via a two-step addition synthesis. Quaternary ammonium bromide (C16QAB) was used as a chain extender of isocyanate terminated PU-*co*-PFPE to form the final polymer (Scheme 1). The amount of C16QAB added (0.12 mmol) was determined based on the typical use concentration of quaternary ammonium compounds in food industry sanitizers [40]. Polymer coatings were deposited into custom fabricated PTFE molds to form free standing 1 cm × 1 cm films used for ATR-FTIR, SEM, and NMR. Polymer was also coated onto 1 cm × 1 cm 304 stainless steel coupons using a custom fabricated device in which stainless steel coupons fit in shallow squares and the polymer is drawn down the surface of the coupons.

### 3.2. Characterization

**Figure 1:**
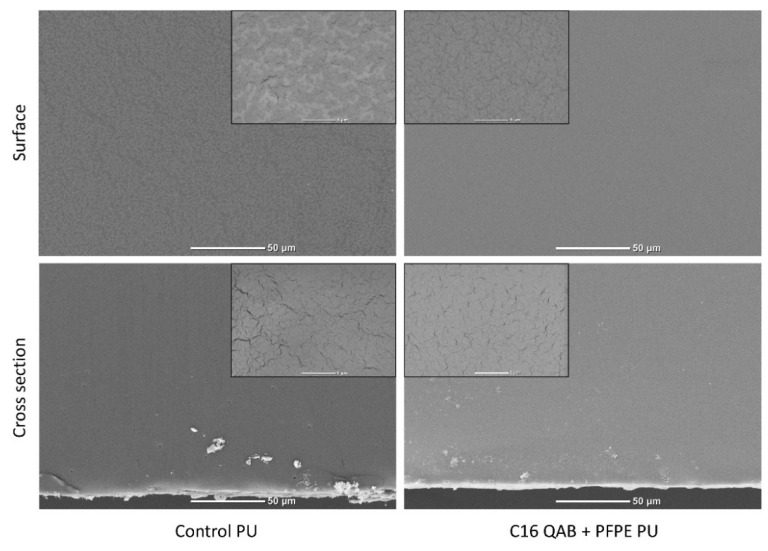
Surface (**top**) and cross section (**bottom**) scanning electron micrographs of control PU (**left**) and C16 QAB + PFPU PU (**right**). Micrographs were obtained at magnifications of ×500 and ×5000 (insets). Images were selected from 5 micrographs acquired across each of 2 independent films.

Scanning electron microscopy revealed homogeneous, smooth surface topography in both control and C16 QAB + PFPU polyurethanes (Figure 1). Uniformly distributed, submicron pores and channels were evident in the cross-sectional micrographs, with no difference in cross-sectional morphology between the polyurethanes. These micrographs indicate that the introduction of the antimicrobial and hydrophobic blocks in the polyurethane do not interfere with coating integrity and uniformity, an important parameter for end-use applications of the coatings.

**Figure 2:**
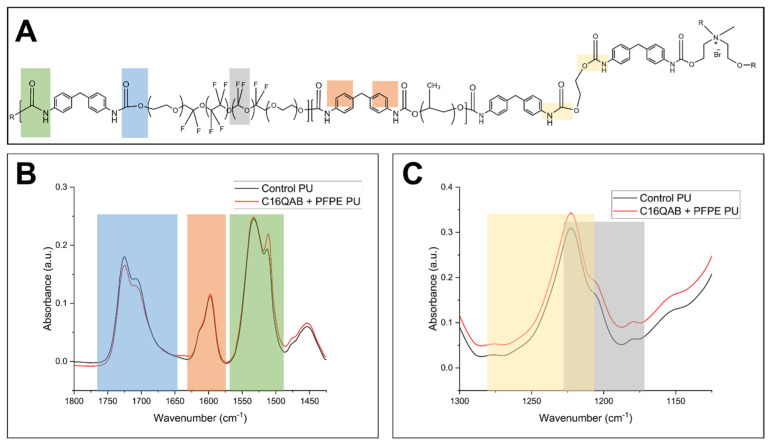
ATR-FTIR spectra of control PU and C16QAB + PFPE PU. (**A**) Chemical structure with characteristic bonds highlighted. (**B**) Urethane region with blue highlighting the ester stretch, orange highlighting cyclic alkyl stretch, and green highlighting CHN bend. (**C**) Yellow highlighting C-O and C-N stretching and fluorocarbon region with gray highlighting CF_2_ band. Spectra displayed were randomly chosen and additional replicate measurements are available upon request.

**Figure 3:**
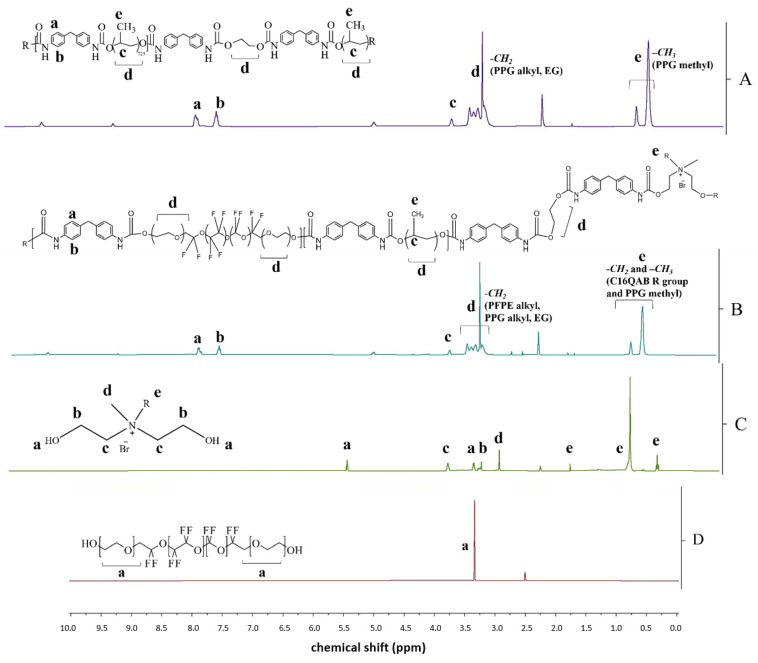
^1^H NMR spectra of synthesized control polyurethane (**A**), C16QAB + PFPE polyurethane (**B**), C16QAB (**C**), and PFPE (**D**). Spectra collected in DMSO-d6 (500 MHz). Lower case letters in chemical structures correspond to resonances (ppm) of the same lower case letter for each polyurethane variant.

Surface chemistry characterization of control polyurethane (control PU) and polyurethane modified with perfluoropolyether and quaternary ammonium bromide (C16QAB + PFPE PU) was performed using ATR-FTIR (Figure 2). Control PU and C16QAB + PFPE PU exhibited N-C=O urethane stretching at 1525 cm^−1^, typical of the base chemistry of polyurethane coatings, and C=O ester stretching created by the reaction with isocyanate and PPG, EG, or PFPE found at 1725 cm^−1^ (Figure 2B). These bands are consistent with the formation of polyurethane as described by Li et al. [41]. The C-F_2_ fluorocarbon stretching is highlighted at 1203 cm^−1^ and no differences were visible between the control PU and the modified PU, likely due to the overlap of C-F_2_ fluorocarbon and C-O ester bands located between 1160 and 1210 cm^−1^. Li et al. discovered similar overlapping in this region of synthesized polyurethane and long chain fluoropolymer coatings [20]. Chemical compositions of control polyurethane, C16QAB + PFPE polyurethane, C16QAB, and PFPE were determined using ^1^H and ^13^C NMR (Figure 3, Figure S1). Perfluoropolyether (Figure 3D) and C16QAB + PFPE (Figure 3B) polyurethane presented resonance centered at 3.3 ppm. However, the ethyl ether group in PFPE is the same group found in ethylene glycol which causes resonance at the same location in the control polyurethane.

XPS surface analysis was used to determine the surface atomic composition of control polyurethane and antimicrobial functionalized C16QAB + PFPE polyurethane (Table 2). Survey scans of C16QAB + PFPE PU confirmed the incorporation and surface orientation of PFPE by the increase in atomic percentage (at.%) fluorine from 0.8 at.% in control polyurethane to 38.0 atomic percent at.% in C16QAB + PFPE PU. The ratio of carbon to fluorine decreases with the addition of PFPE which is expected due to the addition of a heavily fluorinated polyol. The presence of fluorine confirms that polymer blocks containing fluorocarbons are surface oriented which will impart surface hydrophobicity and further reduce surface tension. The ratio of carbon to oxygen also decreases which was expected with the incorporation of an oxygen rich poly(ether). Synthesis of the C16QAB + PFPE PU included greater mmol of nitrogen containing isocyanate along with nitrogen containing C16QAB, which corresponds to the lower ratio of carbon to nitrogen.

Surface hydrophobicity was determined using dynamic sessile drop with needle insertion analysis. The advancing water contact angle of C16QAB + PFPE PU, unmodified PU, and stainless steel are statistically significant from one another with C16QAB at 129.5° ± 4.8, control PU at 95.2° ± 4.6, and stainless steel at 76.3° ± 17.6 (Table 3). Both control polyurethane and functionalized polyurethane possess hydrophobic surfaces (θ > 90°). The addition of the fluoropolymer improved the surface hydrophobicity compared to other antibacterial polyurethanes. Bakhshi et al. created antibacterial polyurethane coatings which displayed a less hydrophobic surface, with all water contact angles under 83° [14]. The polymer produced herein displays the surface effects of the incorporated fluoropolymer and is comparable to other synthesized fluorinated polyurethanes. Wang et al. created a fluorinated polyurethane with improved water contact angles (108°) attributed to the incorporation of fluoropolymers [19]. Gu et al. reported a water contact angle of 118° for synthesized fluorinated polyurethane using 2.4 wt% commercially available Fluorolink E10H diol [29]. The receding contact angles are low, leading to high contact angle hysteresis. This could be attributed to surface-oriented fluorine groups as Yuan and Lee reported high contact angle hysteresis attributed to restriction of water droplet movement caused by hydrophobic surface domains [42].

The critical surface tension of a material is defined by the surface tension of a liquid which can fully spread across the material surface (θ = 0, cos θ = 1) [36,37,43]. The critical surface tensions (γ_cr_) of control polyurethane and modified polyurethane were determined using the Zisman plot method [36]. This method is best suited for low surface tension and non-polar materials and typically uses three to five liquids with known critical surface tensions. The cosine of the advancing contact angle (θ) of each selected liquid on the tested surface was plotted against the known surface tension of the corresponding liquid. Linear fitting is common with the Zisman approach; however, fluoropolymers are better suited for non-linear models as Fox and Zisman reported parabolic fits for tetrafluoroethylene polymers [44]. For this experiment, a non-linear second order polynomial (quadradic) model was applied to the plot and cos θ = 1 intersect was calculated using the non-linear regression equation. In this study, contact angle values were taken using water (72.8 mN m^−1^), glycerol (63.4 mN m^−1^), ethylene glycol (47.7 mN m^−1^), and hexane (18.4 mN m^−1^) on uncoated steel, control polyurethane, and C16QAB + PFPE modified polyurethane. The critical surface tensions of synthesized polyurethanes and stainless steel are presented in Table 4 (Zisman plot presented in Figure S2). Based on this method, C16QAB + PFPE PU possessed low critical surface tension at 13.14 mN m^−1^, whereas control polyurethane had a critical surface tension of 18.07 mN m^−1^. The critical surface tension of type 204 2B finish stainless steel was determined to be 19.96 mN m^−1^. Coating of control polyurethane on the surface of stainless steel did not affect the critical surface tension. Santos et al. reported a critical surface tension for type 316 2B finish stainless steel of 52.5 mN m^−1^ [45]. As far as the authors have found, 13.75 mN m^−1^ is among the lowest reported critical surface tensions for functionalized polyurethane materials with Erceg et al. reporting a surface tension of polydimethylsiloxane-based polyurethane at 13.23 mN m^−1^ [22,26,27,46,47]. However, many reported critical surface tensions differ from one another due to differences in advancing contact angle methodology and critical surface tension methodology. A few methods for determining the critical surface tension include the Owens–Wendt (OWRK), acid-base, and Zisman plotting. Zisman plotting determines the critical surface tension by using multiple liquids compared to OWRK which determines the surface free energy using water and diiodomethane. The reported low critical surface tension is likely due to the surface orientation of the fluorine groups which was observed in XPS surface characterization, as the QAB monomer is not expected to impart any surface changing characteristics. Surface-modified materials have been reported to be favorable in reducing or eliminating bacterial attachment and further biofilm formation due to low surface tension and unfavorable surface chemistries. Khan and others reported correlations with bacterial attachment and material surface free energy with decreased bacterial attachment on low surface energy materials (16 mN m^−1^) [48]. However, when only surface wettability with water (water contact angle) was compared to overall bacterial attachment, no trends were reported [48].

## 4. Antimicrobial Efficacy

**Figure 4:**
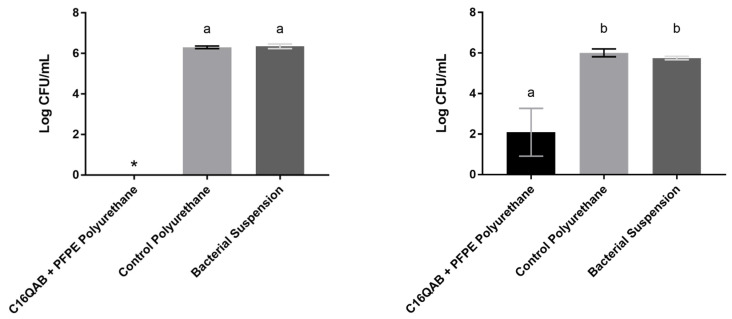
Antimicrobial evaluation results for *Listeria monocytogenes* (**left**) and *Salmonella enterica* (**right**) after 8 h of contact time. Values represent three replicate coupons from two independent replicate experiments (n = 6). Treatments followed by the same letter are not significant (*p* > 0.05). * indicates inactivation below the limit of detection (3 (CFU mL^−1^)).

Antimicrobial efficacy of C16QAB + PFPE PU and unmodified PU was investigated against *Listeria monocytogenes* and *Salmonella enterica* by dynamic contact methods. Coatings were rinsed in 0.3 mM KH_2_PO_4_ at 37 °C while shaking until rinse buffer showed no bacterial inhibition (24 h). Unmodified polyurethane was used as a negative control and a bacterial suspension was used as an experimental control. Eight-hour contact time was chosen due to the shift structuring of food production facilities, with eight hours being a common food production shift followed by cleaning and sanitization. In the *Listeria monocytogenes* experiment, C16QAB + PFPE PU exhibited a log reduction below the limit of detection of the method used (3 CFU mL^−1^). Although buffer directly from the contact tubes was plated (1 × 10^0^ CFU mL^−1^), no colonies were visible and therefore the bacterial concentration was less than 3 CFU mL^−1^. The starting concentration of bacterial suspension placed in the sterile tubes was calculated to be 5 log CFU mL^−1^ based on optical density at 600 nm, however, final concentration in experimentation was 6 log CFU mL^−1^. This discrepancy can be attributed to differences in bacterial growth based on plating and optical density of the preliminary bacterial growth curve. The bacterial suspension concentration for *Listeria monocytogenes* was 6.35 ± 0.13 log CFU mL^−1^ while the bacterial concentration in the polyurethane control tubes was 6.30 ± 0.06 log CFU mL^−1^ (Figure 4) after 8 h. For *Salmonella enterica*, the bacterial suspension concentration in C16QAB + PFPE PU tubes was 2.68 ± 0.18 log CFU mL^−1^ and the bacterial suspension concentration in the polyurethane control tubes was 6.05 ± 0.25 log CFU mL^−1^. Unmodified polyurethane for both experiments was not significantly different than the bacterial suspensions and therefore imparted no antimicrobial activity. The resulting efficacy of C16QAB + PFPE PU can be reported as >6 log or >99.9999% reduction of *Listeria monocytogenes* and >3 log or >99.9% reduction of *Salmonella enterica*. The difference in antibacterial efficacy between Gram-positive *Listeria monocytogenes* and Gram-negative *Salmonella enterica* can be attributed to the differences in cellular structure with Gram-negative bacteria possessing an outer lipid membrane. With the lack of outer lipid membrane, *Listeria monocytogenes* is more susceptible to the long alkyl chains as displayed in the >6 log reduction. Even with the differences in log reduction between Gram-negative and Gram-positive bacteria, the coatings produced herein have provided greater log reduction compared to previously reported functional polyurethanes with quaternary ammonium compounds. Wang et al. synthesized quaternary ammonium bromide functionalized polyurethanes which displayed antibacterial capability between 70.4% and 89.7% against *Escherichia coli, Bacillus subtilis*, and *Staphylococcus aureus* after four hours of contact [16]. Bakhshi et al. synthesized soybean oil-based polyurethanes with quaternary ammonium iodide functionality and reported 83–95% bacterial reduction against *Escherichia coli* and *Staphylococcus aureus* after overnight contact [14]. Direct comparison to other published work is difficult due to differences in methodologies used to determine antimicrobial efficacy; indeed, the wide range of conditions in antimicrobial materials assessments presents a significant and common challenge. Hu et al. used an interesting approach to determine antimicrobial efficacy of their polyurethane adhesives. In addition to the traditional antimicrobial efficacy test (i.e., Kirby–Bauer test), a susceptibility test was performed although the results do not translate to a log reduction value to draw comparisons [15]. Many other researchers utilize the zone of inhibition or Kirby–Bauer test to determine antimicrobial efficacy, however these test methods are not conducive to comparisons of antimicrobial activity (measured in log reduction). With over 6 log reduction of pathogenic *Listeria monocytogenes*, the antimicrobial coatings prepared in this research offer great potential for surface functionalized polyurethane coatings using quaternary ammonium compounds. Future work to explore alternative halogens, such as chlorine, in this polyurethane system could provide interesting information about bacterial susceptibility and coating functionality.

## 5. Conclusions

An antimicrobial polyurethane possessing hydrophobicity and low critical surface tension was prepared via addition polymerization of a commercially available PFPE diol and dihydroxy quaternary ammonium bromide. Surface orientation of fluorine was confirmed by ^1^H NMR, XPS, and by high water contact angle of 129.5° which is associated with tightly packed fluorine polymers and reduced critical surface tension values to only 13.75 mN m^−1^. Incorporation of the quaternary ammonium bromide compound introduced bactericidal properties against both Gram-negative and Gram-positive bacteria. The coating displayed greater than 6 log reduction (>99.9999%) against *Listeria monocytogenes* and greater than 3 log reduction (>99.9%) against *Salmonella enterica.* Future work to explore alternative halogens within the quaternary ammonium compound should be explored to better understand the effect, if any, that the halogen ion possesses. Expanding the range of variables in the antimicrobial activity studies (organisms tested, contact time) represents important future studies to better define the breadth of applications for which the reported material may be suitable. Another interesting path to follow is creating a ‘multi-quat’ antimicrobial polyurethane which incorporates quaternary ammonium compounds with different alkyl chain lengths. Interestingly, both the quaternary ammonium bromide and the perfluoropolyether can be incorporated into other polymers such as polyesters, which require hydroxyl groups for reaction with carboxylic acids. Multifunctional polymers can be synthesized using the techniques discussed herein to create new antimicrobial thermoplastics, foams, and fabrics for use in preventing microbial contamination and biofilm persistence in hospitals, food manufacturing facilities, and biomedical implants.

## Figures and Tables

**Table 1:** Formulation of control polyurethane (PU) and C16QAB + PFPE polyurethane (PU).

	MDI (mmol)	PPG (mmol)	PFPE (mmol)	EG (mmol)	C16QAB (mmol)
Control PU	10.27	6.90	-	4.03	-
C16QAB + PFPE PU	10.35	6.90	0.09	3.22	0.12

**Table 2:** Summary of XPS spectral data including atomic percentage (at.%) utilizing spectral scans of control polyurethane and C16QAB + PFPE polyurethane.

	Carbon (at.%)	Oxygen (at.%)	Nitrogen (at.%)	Fluorine (at.%)	C/O	C/N	C/F
Control PU	72.4	24.7	2.2	0.8	2.9	32.6	91.6
C16QAB + PFPE PU	43.2	17.0	1.8	38.0	2.5	24.7	1.1

**Table 3:** Dynamic water contact angle for stainless steel, control polyurethane, and C16QAB + PFPE polyurethane. Values represent means and standard deviations of two independently synthesized replicates (n = 2). Significant differences between means are indicated by capital letters (Tukey’s HSD, *p* ≤ 0.05).

	Advancing Contact Angle (θ_a_)	Receding Contact Angle (θ_r_)	Hysteresis
Stainless Steel	76.3° ± 17.6 ^A^	18.4° ± 9.6 ^A^	57.9
Control PU	95.2° ± 4.6 ^B^	29.9° ± 6.9 ^B^	65.3
C16QAB + PFPE PU	129.5° ± 4.8 ^C^	28.3° ± 4.3 ^B^	101.2

**Table 4:** Critical surface tensions of stainless steel, control polyurethane, and C16QAB + PFPE polyurethane. Goodness of fit for non-linear second order polynomial regression of Zisman plot data.

	Critical Surface Tension γ_cr_ (mN m^−1^)	Goodness of Fit (r^2^)
Stainless Steel	19.96	0.99
Control PU	18.07	0.99
C16QAB + PFPE PU	13.14	0.99

## Data Availability

Available upon request.

## Supplementary Materials

The following supporting information can be downloaded at: https://www.mdpi.com/article/10.3390/ma16124446/s1, Figure S1: ^13^C NMR spectra. C16QAB (1), PFPE (2), C16QAB + PFPE PU (3), polyurethane (4); Figure S2: Zisman plot of advancing contact angles for four liquids. Second-order polynomial (quadradic) line fitting (dotted). Linear fit (solid).
