# Supplementary material for: Synthesis and Characterization of Antimicrobial Hydrophobic Polyurethane
Source: Materials (Basel). 2023 Jun 17;16(12):4446. doi: 10.3390/ma16124446 (PMC10305346; doi:10.3390/ma16124446)
Supplement: Supplementary file 1 [file materials-16-04446-s001.zip › materials-2384886-supplementary.pdf]

Supplemental Information

Synthesis and characterization of antimicrobial hydrophobic polyurethane for reduction of cross-contamination

Autumn M. RUDLONG[1], Julie M. GODDARD[1*]

[1]Department of Food Science, Cornell University, Ithaca, NY, 14853

*Corresponding author

Telephone: 607-255-8622

Email address goddard@cornell.edu (J.M. Goddard), amr452@cornell.edu (A.M. Rudlong)

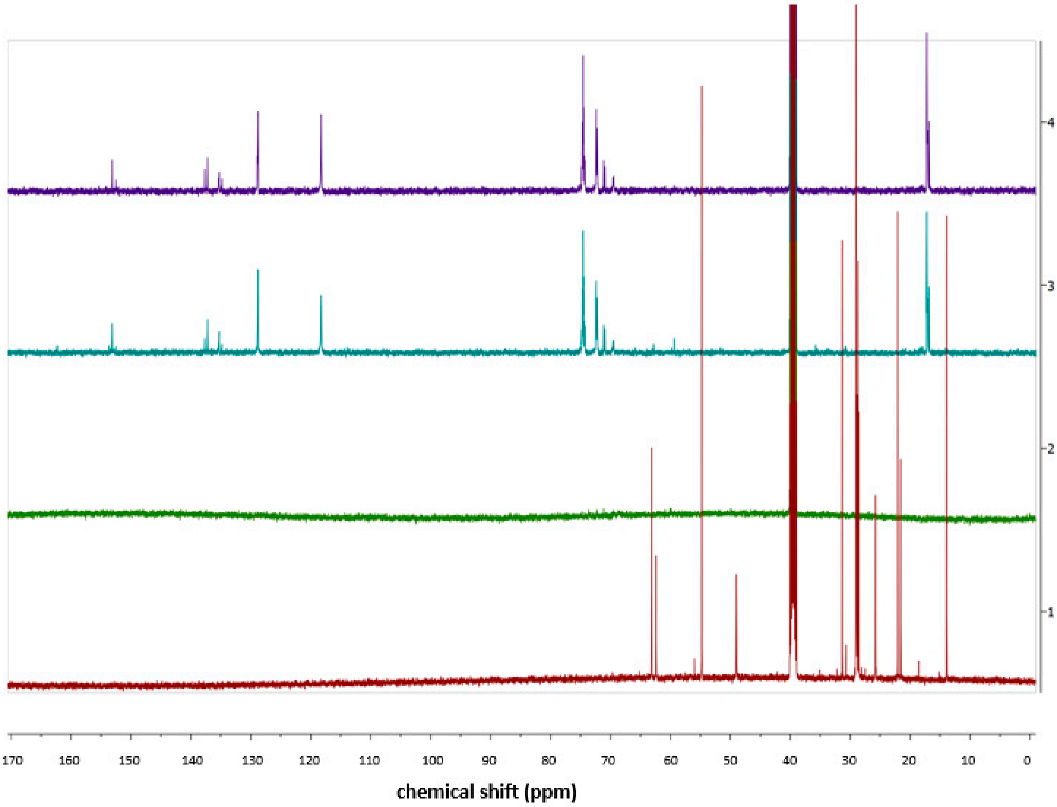

Figure S1. ^{13}C NMR spectra. C16QAB (1), PFPE (2), C16QAB + PFPE PU (3), polyurethane (4).

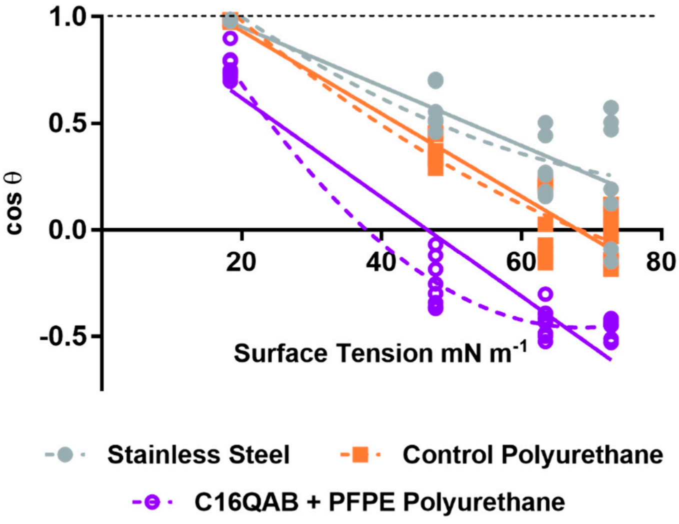

Figure S2. Zisman plot of advancing contact angles for four liquids. Second-order polynomial (quadradic) line fitting (dotted). Linear fit (solid).
